# Alteration of Extracellular Matrix Components in the Anterior Pituitary Gland of Neonatal Rats Induced by a Maternal Bisphenol A Diet during Pregnancy

**DOI:** 10.3390/ijms222312667

**Published:** 2021-11-23

**Authors:** Bumpenporn Sanannam, Sasikarn Looprasertkul, Songphon Kanlayaprasit, Nakarin Kitkumthorn, Tewarit Sarachana, Depicha Jindatip

**Affiliations:** 1 Department of Anatomy, Faculty of Medicine, Chulalongkorn University, 1873 Rama 4 Rd., Wangmai, Pathumwan, Bangkok 10330, Thailand; bumpenporn.s@student.chula.ac.th (B.S.); sasikarn.loo@alumni.chula.ac.th (S.L.); 2 Department of Anatomy, Division of Histology and Cell Biology, School of Medicine, Jichi Medical University, 3311-1 Yakushiji, Shimotsuke 329-0498, Tochigi, Japan; 3 Department of Clinical Chemistry, Faculty of Allied Health Sciences, Chulalongkorn University, Bangkok 10330, Thailand; songphon.ka@student.chula.ac.th; 4 Department of Oral Biology, Faculty of Dentistry, Mahidol University, Payathai Rd., Ratchathewi, Bangkok 10400, Thailand; nakarin.kit@mahidol.ac.th; 5 Age-Related Inflammation and Degeneration Research Unit, Department of Clinical Chemistry, Faculty of Allied Health Sciences, Chulalongkorn University, 154 Rama 1 Rd., Wangmai, Pathumwan, Bangkok 10330, Thailand; tewarit.sa@chula.ac.th; 6 Systems Neuroscience of Autism and Psychiatric Disorders (SYNAPS) Research Unit, Department of Clinical Chemistry, Faculty of Allied Health Sciences, Chulalongkorn University, Bangkok 10330, Thailand

**Keywords:** anterior pituitary gland, bisphenol A, collagen, endocrine-disrupting chemicals, extracellular matrix, folliculostellate cell, matrix metalloproteinase, neonate, pericyte, tissue inhibitors of metalloproteinase

## Abstract

The extracellular matrix (ECM) plays crucial roles in the anterior pituitary gland via the mechanism of cell–ECM interaction. Since bisphenol A (BPA), a well-known endocrine disruptor, can cross through the placenta from mother to fetus and bind with estrogen receptors, cell populations in the neonatal anterior pituitary gland could be the target cells affected by this chemical. The present study treated maternal rats with 5000 µg/kg body weight of BPA daily throughout the pregnancy period and then investigated the changes in ECM-producing cells, i.e., pericytes and folliculostellate (FS) cells, including their ECM production in the neonatal anterior pituitary at Day 1. We found that pericytes and their collagen synthesis reduced, consistent with the increase in the number of FS cells that expressed several ECM regulators—matrix metalloproteinase (MMP) 9 and the tissue inhibitors of metalloproteinase (TIMP) family. The relative *MMP9/TIMP1* ratio was extremely high, indicating that the control of ECM homeostasis was unbalanced. Moreover, transmission electron microscopy showed the unorganized cell cluster in the BPA-treated group. This study revealed that although the mother received BPA at the “no observed adverse effect” level, alterations in ECM-producing cells as well as collagen and the related ECM balancing genes occurred in the neonatal anterior pituitary gland.

## 1. Introduction

Bisphenol A (BPA) is acknowledged as one of the most common endocrine-disrupting chemicals and is able to bind to estrogen receptors [1]. BPA is primarily used as a monomer of polycarbonate plastics and epoxy resin production found in many consumer products and can cause food contact and contamination [2]. Since BPA has the potential to transfer through umbilical blood circulation and fetal metabolization of this chemical is limited, this chemical is detected at a high concentration in both the placenta and fetus [3]. Exposure to BPA during the embryonic stage was mentioned in the decline of reproductive ability in adulthood, abnormal estrus cycles, and decreases in LH serum [4,5]. Although these studies did not directly investigate the hypothalamo-hypophyseal level, the results indicate that the anterior pituitary gland is also a BPA target tissue.

The anterior pituitary gland is a major organ that plays an important role in the endocrine system. This gland produces many essential hormones related to body homeostasis. Cell populations of the anterior pituitary gland are composed of five types of hormone-producing cells and nonhormone-producing cells, i.e., folliculostellate (FS) cells, endothelial cells, pericytes, novel desmin-immunopositive perivascular cells and macrophages [6]. In addition to cell populations in the pituitary gland, the extracellular matrix (ECM) diffuses in the areas around cell clusters and the perivascular space [7], and our previous studies found that Collagen types I and III in the rat anterior pituitary gland are produced mainly by pericytes facilitated by FS cells [8,9]. Moreover, these two cell types expressed some of the matrix metalloproteinase (MMP) and tissue inhibitors of metalloproteinase (TIMP) genes that maintain ECM equilibrium [10,11].

The effects of BPA on the alteration of ECM components and characteristics have been documented in some organ conditions, such as liver fibrosis and cardiovascular function [12,13]. In the anterior pituitary gland, the adverse effects of BPA on hormone-producing cells and their secretion have been reported in several studies. Publications have revealed that prenatal BPA administration at doses up to the “no observed adverse effect” level (NOAEL) is related to increases in the proliferation of progenitor cells and gonadotrophs, as well as the mRNA levels of the gonadotropin-releasing hormone receptor, follicle-stimulating hormone and luteinizing hormone [14,15]. In addition, other studies have reported the elevation of fetal serum thyroid-stimulating hormone secretion and the reduction of growth hormones in offspring in response to BPA exposure [16,17]. However, BPA-induced ECM changes, especially in the anterior pituitary of neonates, have not been investigated. Therefore, the present study performed immunohistochemistry to investigate the number and characteristics of ECM-producing cells, i.e., pericytes and FS cells, including the distribution and intensity of collagen staining. Gene expression of collagen Type I and III, MMP9 and the TIMP family produced by these ECM-producing cells was evaluated. Moreover, transmission electron microscopy revealed cell organization in the anterior pituitary gland of both control and BPA-treated neonatal rats.

## 2. Results

### 2.1. Effects of BPA at the NOAEL Level on ECM-Producing Cells

**Figure 1:**
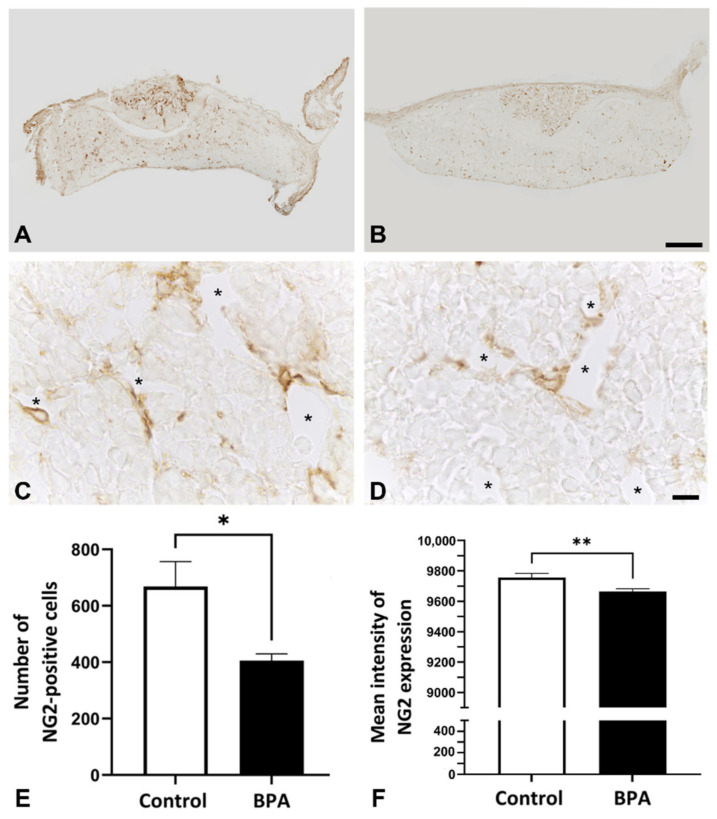
Immunohistochemistry of NG2 on postnatal Day 1 in the anterior pituitary glands of control rats ((**A**,**C**): higher magnification view of (**A**)) and BPA-treated rats ((**B**,**D**): higher magnification view of (**B**)). The number (**E**) and mean intensity (**F**) of NG2-positive cells decreased in the BPA-treated group. Note: the capillary lumens (asterisks), * *p* < 0.05, ** *p* < 0.01. Scale bars: 100 µm (**A**,**B**); 10 µm (**C**,**D**).

NG2 was used as a pericyte marker, which was observed in both the anterior and posterior lobes. Focusing on the anterior pituitary lobe, NG2 immunopositive cells were closely related to the capillaries (Figure 1A–D). The number of pericytes and the intensity of immunosignals significantly decreased in the BPA-treated group (405.6 ± 23.96 and 9665 ± 17.87, respectively) compared with the control group (668.0 ± 89.15 and 9756 ± 27.02, respectively) (Figure 1E,F).

**Figure 2:**
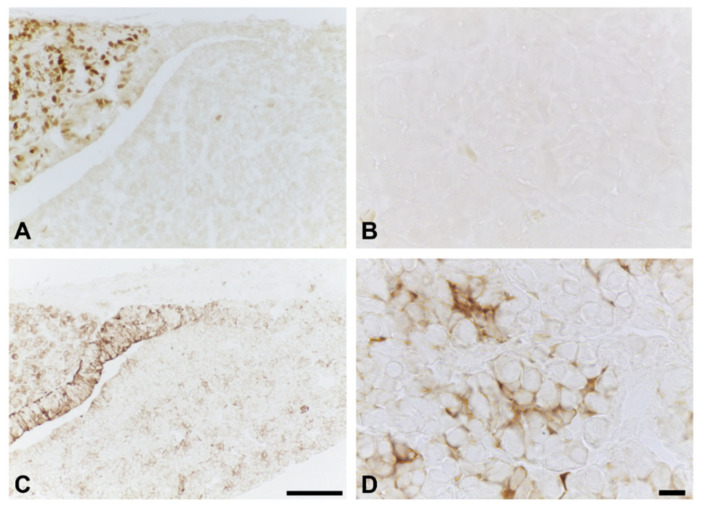
Immunohistochemistry of S100 ((**A**,**B**): higher magnification view of (**A**)) and aldolase C ((**C**,**D**): higher magnification view of (**C**)) on postnatal Day 1 in the anterior pituitary glands of control rats. Scale bars: 100 µm (**A**,**C**); 10 µm (**B**,**D**).

**Figure 3:**
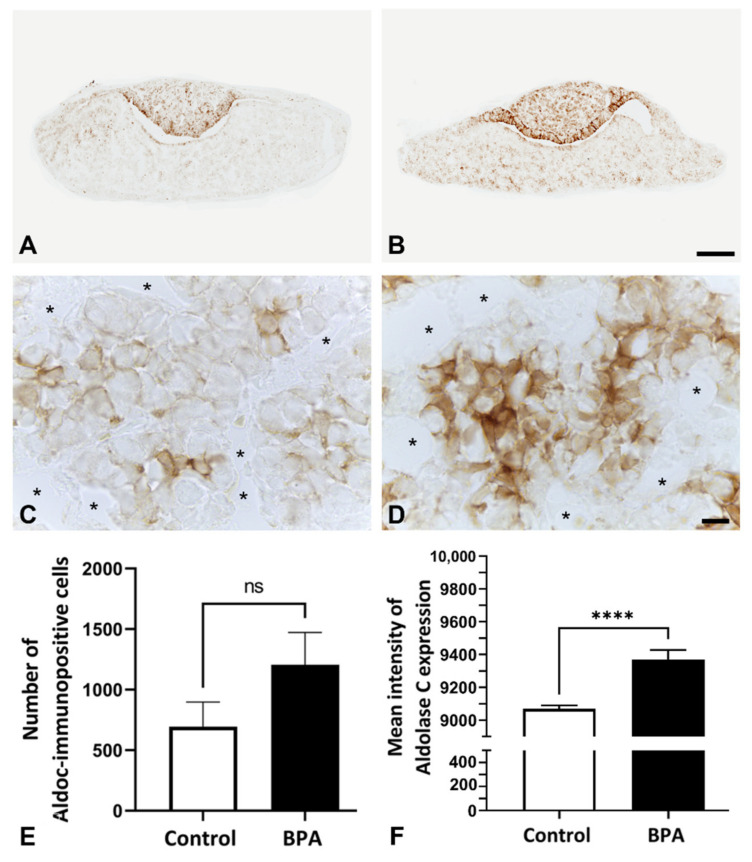
Immunohistochemistry of aldolase C on postnatal Day 1 in the anterior pituitary glands of control rats ((**A**,**C**): higher magnification view of (**A**)) and BPA-treated rats ((**B**,**D**): higher magnification view of (**B**)). Number (**E**) and mean intensity (**F**) of aldolase C immunopositive cells in the control and BPA-treated groups. Note: the capillary lumens (asterisks). No significant differences (ns), **** *p* < 0.0001. Scale bars: 100 µm (**A**,**B**); 10 µm (**C**,**D**).

As pericytes and FS cells interact with each other for ECM production in the anterior pituitary gland, the number of FS cells was also examined by immunohistochemistry. Anti-S100 antibody is commonly used as a marker of FS cells in several adult rodents. However, immunoreactivity could not be observed in the anterior lobe of rats at postnatal Day 1 (Figure 2A,B). Therefore, aldolase C, a novel FS cell marker, was selected for FS cell identification instead of S100. The preliminary results showed that FS cells were intensely stained by anti-aldolase C antibody in the intact neonatal anterior pituitary glands (Figure 2C,D). In the present study, increases in the number of aldolase C immunopositive cells and immunoreactive intensity were observed in the BPA-treated group (1206 ± 266.5 and 9368 ± 58.62, respectively) compared with the control group (694.8 ± 202.5 and 9068 ± 20.84, respectively) (Figure 3).

### 2.2. Alterations of ECM Components Focusing on Collagens

**Figure 4:**
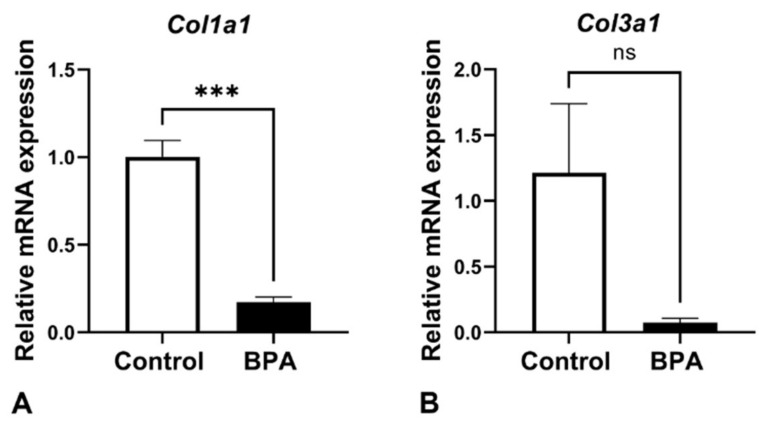
*Col1a1* (**A**) and *col3a1* (**B**) gene expression in the control and BPA-treated groups. Note: no significant differences (ns), *** *p* < 0.0001.

Collagen Types I and III were examined by using RT-qPCR analysis. The mRNA expression of both *col1a1* and *col3a1* was downregulated in the BPA-treated group. In particular, collagen Type I showed a significant decrease in the BPA-treated group compared with the control (Figure 4).

**Figure 5:**
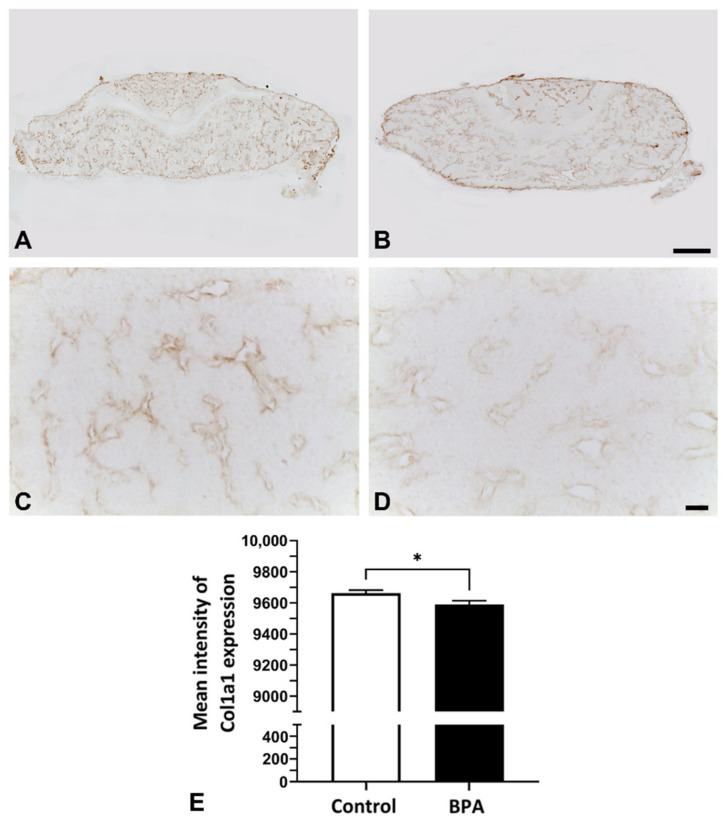
Immunohistochemistry of collagen Type I on postnatal Day 1 in the anterior pituitary glands of control rats ((**A**,**C**): higher magnification view of (**A**)) and BPA-treated rats ((**B**,**D**): higher magnification view of (**B**)). Mean intensity in the control and BPA-treated groups (**E**). Note: * *p* < 0.05. Scale bars: 100 µm (**A**,**B**); 10 µm (**C**,**D**).

In addition, the immunohistochemistry of collagen Type I in the gland was also observed under light microscopy. The results showed that the pattern of collagen distribution in BPA rats seemed to be less than the pattern of collagen distribution in the control rats (Figure 5A–D). The immunoreactive intensity of collagen was decreased in the BPA group (Figure 5E).

### 2.3. Alterations of ECM Balancing Regulators

**Figure 6:**
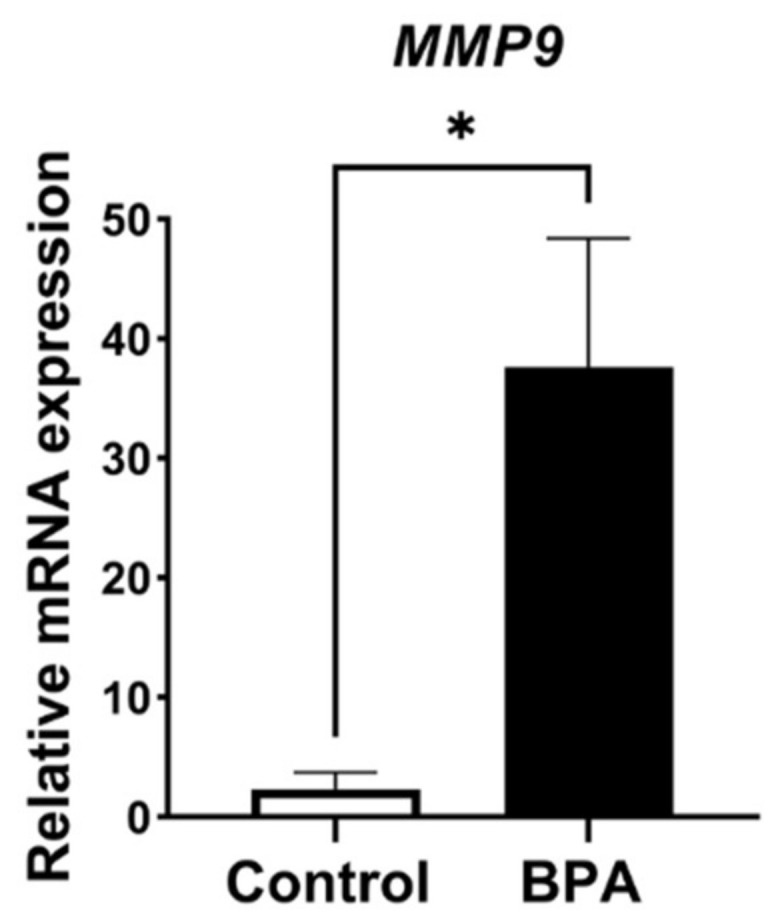
*MMP9* mRNA expression in the control and BPA-treated groups. Note: * *p* < 0.05.

**Figure 7:**
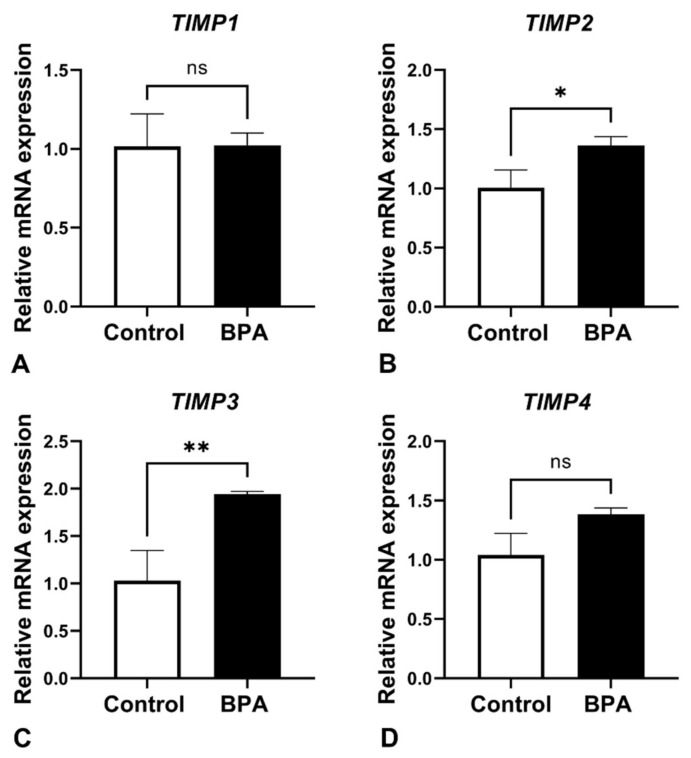
*TIMP 1* (**A**), *TIMP 2* (**B**), *TIMP3* (**C**) and *TIMP 4* (**D**) gene expression in the control and BPA-treated groups. Note: no significant differences (ns), * *p* < 0.05 and ** *p* < 0.01.

**Figure 8:**
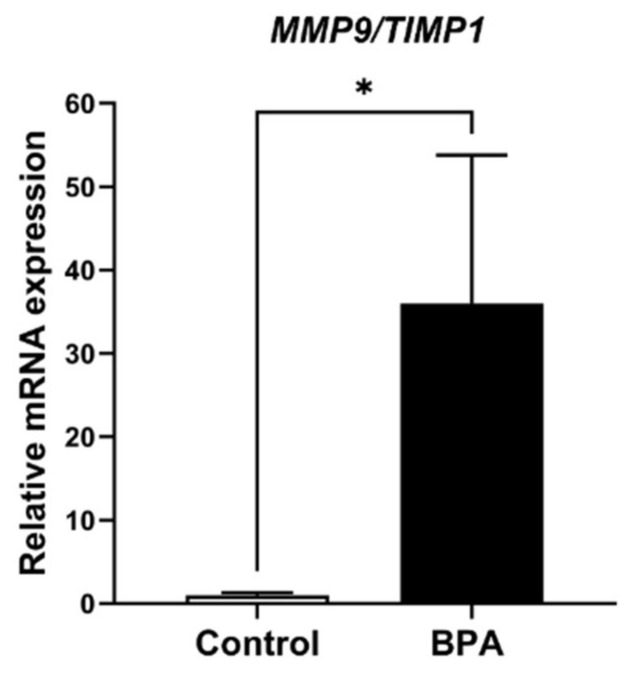
Relative *MMP9*/*TIMP1* ratio of mRNA expression in the control and BPA-treated groups. Note: * *p* < 0.05.

ECM balancing, in both general and pathological conditions, is involved in MMP and TIMP interactions. Therefore, MMP9, which is produced by FS cells and is one of the major MMPs in the anterior pituitary gland, was examined. The results revealed that *MMP9* mRNA expression levels showed statistically significant differences (Figure 6). For the TIMP family, mRNA expression of *TIMP1*, *TIMP2*, *TIMP3* and *TIMP4* was also detected in the glands of both the control and BPA-treated groups. The results showed that no difference in *TIMP1* was detectable between the two groups, whereas the expression levels *TIMP2* and *TIMP3* mRNA were upregulated in BPA-treated rats. Note that the mRNA level of *TIMP4* was also increased, but the difference between groups was not statistically significant (Figure 7). In addition, the relative ratio of *MMP9*/*TIMP1* is shown in Figure 8.

### 2.4. Tissue Organization Observed by Transmission Electron Microscopy

**Figure 9:**
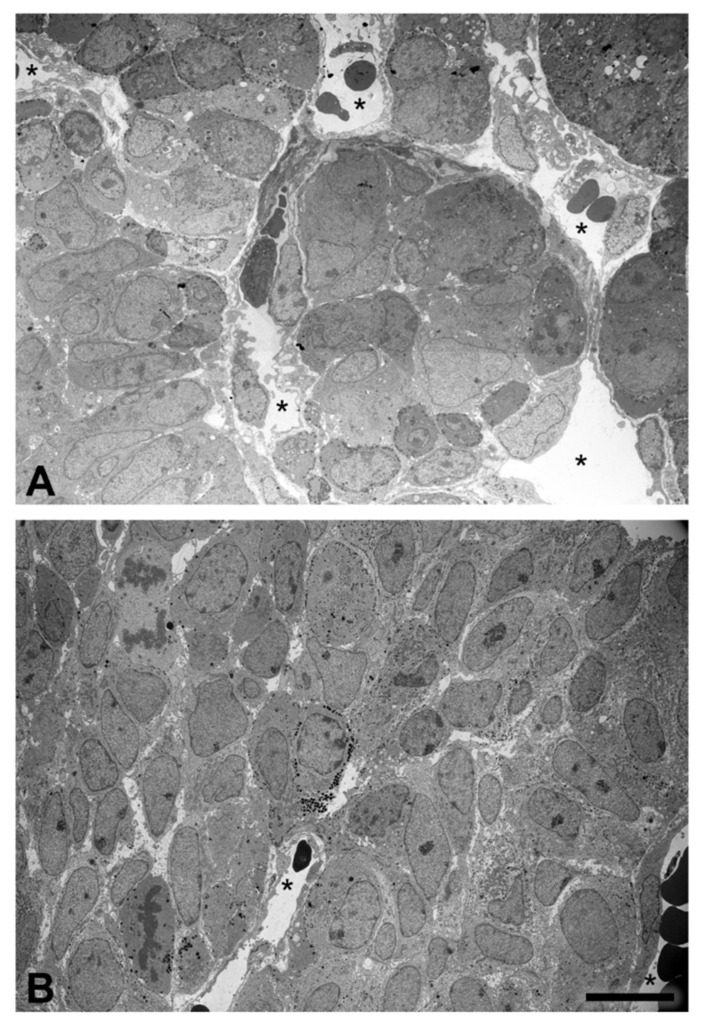
Transmission electron microscopy of cell cluster organization in the neonatal anterior pituitary gland of the control (**A**) and BPA-treated groups (**B**). Note: the capillary lumens (asterisks). Scale bar: 10 µm.

In control rats, the anterior pituitary gland of the neonates demonstrated a cell cluster formation encircled by the fenestrated capillary network, while the cells of BPA-treated rats were not well organized in forming the cluster, as was the case in the control group (Figure 9). The morphology of pericytes and FS cells was also observed, although there was no difference in fine structure between the control and BPA-treated rats.

## 3. Discussion

The present study revealed that maternal BPA exposure affects ECM-producing cells and their activities in the neonatal anterior pituitary gland. The number of pericytes, NG2 immunosignal intensity and collagen immunohistochemical staining decreased, consistent with the decrease in collagen mRNA expression. In addition, increases in FS cell numbers and their staining intensity correlated with alterations in ECM balancing regulators. Moreover, transmission electron microscopic visualization revealed predominantly unformed cluster characteristics in the anterior pituitary gland of prenatal BPA exposure rats.

Pericytes in the anterior pituitary gland are known as synthesizers of collagen Types I and III from postnatal development to adulthood [8,18]. Our previous study demonstrated that the collagen activity of pituitary pericytes was downregulated in prolactinoma rats that were treated with diethylstilbestrol (DES) for 3 months [19]. The present study revealed the suppressive effect of maternal BPA exposure on collagen expression and the number of pericytes in neonatal rats (Figure 1, Figure 4 and Figure 5). There has been a report of the similarity in chemical structure between DES and BPA, showing that they are classified as estrogen-like endocrine-disrupting chemicals and can bind to estrogen receptors that induce hyperprolactinemia [20]. As pericytes also contain estrogen receptors [21], we presume that the effects of BPA on collagen suppression occur via this pathway. Although BPA can induce prolactinoma with high proliferation of several cell types [22], our present study used the NOEAL of BPA, which did not reach the pathological angiogenesis of tumors. Therefore, the number of vascular pericytes in this study was not high, as is the case with DES administration. However, a small number of pericytes resulting in collagen reduction is reasonable.

A specific marker that is commonly used for detecting FS cells in the anterior pituitary gland is S100. In our preliminary study, S100 staining was not observed in any area on Day 1 of age in rats (Figure 2A,B). This result resembled the study of Wada et al. (2014), which reported that S100 immunopositive signals were first identified on the anterior pituitary on around postnatal Day 15 [23]. Aldolase C was recently reported as a novel marker of FS cells in adult mouse pituitary glands [24]. Interestingly, the present study succeeded in identifying FS cells in the newborn rat pituitary by using this anti-aldolase C antibody (Figure 2C,D). We hypothesized that the differential protein marker expression is due to the highly dynamic characteristics of FS cells during pituitary development. Therefore, this finding could help researchers investigate FS cells at an early postnatal stage through to adulthood in rat strains under conventional light microscopic levels.

Since FS cells also present estrogen receptors [25], endocrine-disrupting chemicals could induce changes in the properties of these cells. In the present study, BPA had significant positive effects on the cell number and immunostaining intensity of FS cells in contrast to pericytes. Brannick et al. (2012) mentioned that the use of low BPA doses in mice during the prenatal stage resulted in an increase in the cell proliferation of gonadotrophs and SOX2-expressing progenitor cells in the neonatal anterior pituitary gland [14]. Several publications have reported the expression of progenitor or stem cell markers in FS cells, including SOX2 [26,27,28]. These data suggest that FS cells in our present study are also one of the proliferating cell populations that increase in number and activity in prenatal BPA-treated offspring. In addition, it is possible that the alterations in FS cells might involve other indirect adverse effects, such as local hormones in the gland, since FS cells present several pituitary hormone receptors [29]. For example, Ahmed (2016) revealed serum TSH elevation in 20-day-old fetuses after treating maternal rats with BPA throughout gestation Days 1 to 20 [16]. Similar to the study from Brokken et al. (2005), they found that the FS cell line contained TSH receptors, and these cells expressed several cell proliferation-related genes that were regulated by TSH [30]. However, the level of TSH secretion induced by BPA remains controversial. Brannick et al. (2012) reported that *TSH* mRNA in thyrotrophs was not changed in postnatal Day 1 mice exposed to maternal BPA during gestation Days 10.5 to 18.5 [14]. Therefore, the effects of local pituitary hormones on FS cells in response to BPA exposure need to be clarified in further studies.

Pituitary pericytes require a paracrine factor, TGFβ2, to be released from FS cells to synthesize collagens [9]. In addition, the ECM proteolytic enzyme and ECM proteolytic enzyme inhibitors were detected in either pericytes or FS cells, i.e., MMP9 and the TIMP family. Ilmiawati et al. (2012) demonstrated that FS cells on laminin expressed *MMP9*, and then their MMP9 returned to promote the proliferation of FS cells themselves [10]. These data support our present findings of numerous FS cells (Figure 3) and a significant elevation of *MMP9* levels (Figure 6). Thus, autocrine stimulation would be considered an additional route of FS cell proliferation triggered by BPA. ECM turnover is regulated via MMP–TIMP interactions in rat and human anterior pituitary glands. There are four subtypes of the TIMP family that act as tissue inhibitors of MMPs. A publication reported that *TIMP1*, *TIMP2* and *TIMP3* mRNAs were expressed mainly in FS cells, whereas *TIMP4* was not detectable in any cells of the anterior pituitary gland of adult rats [11]. Surprisingly, we found the expression of all *TIMPs*, including *TIMP4*, in neonatal rats of both the control and BPA-treated groups. However, TIMP4-producing cells were not investigated in the present study. Although all *TIMPs* in the BPA-treated group tended to increase to regulate ECM degradation (Figure 7), the *MMP* level remained high (Figure 6). We hypothesized that even though the expression of TIMPs was elevated, it was not high enough to inhibit the increasing level of MMPs. Moreover, the concept of the MMP/TIMP ratio, which demonstrates ECM equilibrium, might be useful for explaining this phenomenon [31]. In Figure 8, the high relative *MMP9*/*TIMP1* ratio, which might occur with other *MMPs*/*TIMPs*, in the BPA-treated group could indicate an ECM imbalance. Moreover, transmission electron microscopy (Figure 9) showed that BPA also affected cell cluster formation in the anterior pituitary gland.

## 4. Materials and Methods

### 4.1. Animal and Treatment

Wistar rats at 8 weeks of age were purchased from the National Laboratory Animal Center (NLAC), Thailand. Animals were housed under standard temperature (21 ± 1 °C) and humidity conditions (30–70%) with a 12-h light/dark cycle at Chulalongkorn University Laboratory Animal Center. After normal inbreeding, maternal rats were divided into 2 groups. The amount of daily treated BPA was calculated from the maternal weight each day. The rats were given food and RO-UV water ad libitum. These rats were given 5000 µg/kg BW of BPA daily [32,33,34,35] (Cat. No. 239658, Sigma–Aldrich, St. Louis, MO, USA) in absolute ethanol (Cat. No. 1000983, Merck Millipore, Darmstadt, Germany) and corn oil by oral administration throughout their pregnancy periods (approximately 21 days) as described in our previously published study [33]. This dose was selected based on the “no observed adverse effect” level (NOAEL) for BPA determined by the US Food and Drug Administration (FDA) and the European Food Safety Authority (EFSA). A mixture of absolute ethanol with corn oil was applied as the vehicle control treatment. All animal experimental procedures and research protocols were approved by the Chulalongkorn University Animal Care and Use Committee (Animal Use Protocol No. 1773011) and the Animal Ethical Committee of the Faculty of Medicine (010/2562), Chulalongkorn University, Bangkok, Thailand.

### 4.2. Tissue Collection

After parturition, postnatal Day 1 rats were euthanized by sodium pentobarbital via intraperitoneal injection. The rats were then decapitated, followed by freezing on ice. The pituitary gland was promptly removed with the aid of a stereomicroscope (SMZ800 Zoom; Nikon, Japan).

### 4.3. Immunohistochemistry

Five neonatal pituitary glands from each group were immediately immersed in 4% PFA in 0.1 M PB (pH 7.4) overnight, followed by 30% sucrose (Cat. No. S0111, TCI, Tokyo, Japan) in 0.1 M PB (pH 7.4) for 48 h. Glands were then embedded in Tissue-tek (Cat. No. 4583, Sakura Finetechnical, Tokyo, Japan) and stored at −80 °C. Frontal 6 µm cryosections were cut by a cryostat (CM1950; Leica Biosystems, Nussloch, Germany) for immunohistochemistry. To block nonspecific signals, sections were incubated in phosphate-buffered saline (PBS) containing 2% normal goat serum (Cat. No. S-1000, Vector Laboratories, Burlingame, CA, USA). Anti-NG2 rabbit polyclonal antibody (pericyte marker, diluted 1:600; Cat. No. AB5320, Merck Millipore, Darmstadt, Germany), anti-S100 rabbit polyclonal antibody (FS cell marker, diluted 1:1000; Cat. No. Z0311, Agilent DAKO, Santa Clara, CA, USA), anti-aldolase C rabbit polyclonal antibody (novel FS cell marker, diluted 1:200; Cat. No. AB_2571658, Frontier Institute Co., Ltd., Hokkaido, Japan) or anti-collagen type I rabbit polyclonal antibody (diluted 1:400; Cat. No. ab34710, Abcam, Cambridge, UK) were incubated overnight at room temperature. After primary antibody incubation, tissues were incubated in biotinylated anti-rabbit IgG (diluted 1:150; Cat. No. BA-1000, Vector Laboratories, Burlingame, CA, USA) at 30 °C for 30 min. The ABC method (Cat. No. PK-4000, Vector Laboratories, Burlingame, CA, USA) and 3,3′-diaminobenzidine as the substrate (Cat. No. D5637, DAB; Dojindo Laboratories, Kumamoto, Japan) were applied to demonstrate the signal expression. Five random fields at 40× magnification were captured by light microscopy (DM1000; Leica Microsystems, Wetzlar, Germany). The number of immune-positive cells and the immunostaining intensity were evaluated by ImageJ software version 1.52a (Wayne Rasband, National Institutes of Health, Bethesda, MD, USA) and ZEN software version 3.0 (Blue edition, Carl Zeiss Microscopy, Oberkochen, Germany).

### 4.4. Determination of mRNA Expression by RT-qPCR

Three rats per group were sacrificed for this experiment. First, the posterior pituitary lobe was removed during dissection. The anterior pituitary tissues were then quickly put into a tube containing RNAlater (AM7024, Thermo Fisher Scientific Inc., Rockford, IL, USA). These glands were stored at −80 °C until use. Total RNA of the anterior pituitary gland was isolated by using TRIzol’s protocol (Cat. No. 15596026, Invitrogen, Carlsbad, CA, USA). Next, cDNA synthesis was performed by using a RevertAid First-Strand cDNA Synthesis Kit (Cat. No. K-1621, Thermo Fisher Scientific Inc., Rockford, IL, USA) according to the manufacturer’s protocol. The stock of cDNA was stored at 25 ng/µL. One microliter of the 5 ng/µL cDNA template was used for qPCR analysis by mixing with 2X Greenstar Master Mix (Cat. No. K-6253, Bioneer, Daejeon, Korea), both forward and reverse primers, and ultrapure water. The mixture was then incubated in a CFX 96 thermocycler (Bio–Rad Laboratories, Hercules, CA, USA). Amplification was performed according to these steps: an initial denaturing step at 95 °C for 15 min, followed by 45 cycles of 10 s, 95 °C as the denaturing step and annealing/extension for 30 s at 55 °C. Melting curve data were considered to confirm product formation (65 to 95 °C). The expression was calculated using the 2^−∆∆Ct^ method; 18S ribosomal RNA (*RN18S*) was used as the internal control. Primers for PCR (Table 1) were explored by using the USCS Genome Browser (https://genome.ucsc.edu./ (accessed on 10 June 2019)) and Ensembl (https://asia.ensembl.org/inderx.html (accessed on 10 June 2019)). The sequences of primers were created by Primer3 software (http://bioinfo.ut.ee/primer3-0.4.0/ (accessed on 10 June 2019)).

### 4.5. Transmission Electron Microscopic (TEM)

TEM was performed to evaluate the changes in cell cluster organization. Three collected pituitary glands per group were fixed with 2% glutaraldehyde (Cat. No. 16220, EMS, Hatfield, PA, USA) in 0.1 M PB (pH 7.4) for 2 h, followed by 4 washes with PB. Next, tissues were treated with 1% osmium tetroxide (OsO_4_; Cat. No. 19110, EMS, Hatfield, PA, USA) on ice for 90 min. After postfixation in OsO_4_, specimens were washed with cold distilled water, dehydrated by an alcohol series (Cat. No. 1000983, Merck Millipore, Darmstadt, Germany) and propylene oxide (Cat. No. 807027, Merck Millipore, Darmstadt, Germany). Next, specimens were embedded in epoxy resin and polymerized at 60 °C. The specimen resin blocks were cut into ultrathin sections at 70 nm thickness by an ultramicrotome (EM UC7, Leica, Vienna, Austria) and stained with lead citrate (Cat. No. 178000, EMS, Hatfield, PA, USA) and uranyl acetate (Cat. No. 22400, EMS, Hatfield, PA, USA). Ultrastructural changes in cells and cluster organization were examined under a transmission electron microscope (JEM-1400PLUS; JEOL, Tokyo, Japan).

### 4.6. Statistical Analysis

The results are presented as the mean ± standard error of the mean (SEM). Student’s *t*-test was used to compare differences between groups, which were considered statistically significant at a value of *p* < 0.05. The analyses were calculated using GraphPad Prism 9.2.0 (GraphPad Software, Inc., San Diego, CA, USA) and IBM SPSS Statistics version 23 (SPSS, Inc., Armonk, NY, USA).

## 5. Conclusions

The present study revealed that even though fetuses received BPA at a dose without observed adverse effects, alterations in ECM-producing cell properties and ECM products, as well as related ECM balancing genes and cell cluster formation, were detectable in the anterior pituitary gland. The findings of this study support the hypothesis that the prenatal stage is highly sensitive to endocrine-disrupting chemicals because this stage is one of the critical periods during development. However, the limitations of the present study are the use of a single dose of BPA and one-period evaluation. In addition, there are several types of bisphenols used instead of BPA and labeled on products as BPA-free nowadays. Awareness of the harm of these bisphenols should also be raised. For further directions, the effects of bisphenol derivatives such as BPF or BPS on ECM components in the anterior pituitary gland at various doses and times during gestation until the postnatal period need to be verified. Moreover, the numbers of ECM-related cells in connection with the numbers of hormone-producing cells, as well as their three-dimensional physical interactions, should also be included in the further studies. These data will provide a better understanding of ECM’s controlling function in the anterior pituitary gland under bisphenol derivative administration.

## Figures and Tables

**Table 1:** Primers of the targeted genes.

Genes	Forward Primer (5′>-3′)	Reverse Primer (5′>-3′)
Collagen type 1 (*Col1a1*)	CCTGACGCATGGCCAAGA	CTGGGCAGAAAGGACAGCA
Collagen type 3 (*Col3a1*)	TGCCACCCTGAACTCAAGAG	CACCAGCATATGTCCACCA
Matrix metalloproteinase 9 (*MMP9*)	AGAGCGTTACTCGCTTGGA	CTGCAGGAGGTCATAGGTCA
Tissue inhibitor of metalloproteinase 1 (*TIMP1*)	CTGGCATCCTCTTGTTGCT	AGGTGGTCTCGATGATTTCTG
Tissue inhibitor of metalloproteinase 2 (*TIMP2*)	GACGTTGGAGGAAAGAAGGA	GGCTCTTCTTCTGGGTGATG
Tissue inhibitor of metalloproteinase 3 (*TIMP3*)	TGGGAAAGAAGCTGGTGAA	CACATGGGGCATCTTACTGA
Tissue inhibitor of metalloproteinase 4 (*TIMP4*)	CACGCCATTTGACTCTTCTC	CTCCCAGGGCTCAATGTAGT
18S Ribosomal RNA (*RN18S*)	CTGGATACCGCAGCTAGGAA	GAATTTCACCTCTAGCGGCG

## Data Availability

Not applicable.
